# Brain MRI Reveals Ascending Atrophy in Parkinson's Disease Across Severity

**DOI:** 10.3389/fneur.2019.01329

**Published:** 2019-12-18

**Authors:** Jamie C. Blair, Matthew J. Barrett, James Patrie, Joseph L. Flanigan, Scott A. Sperling, W. Jeffrey Elias, T. Jason Druzgal

**Affiliations:** ^1^Department of Radiology and Medical Imaging, University of Virginia Health System, Charlottesville, VA, United States; ^2^Department of Neurology, University of Virginia Health System, Charlottesville, VA, United States; ^3^Department of Public Health Sciences, University of Virginia Health System, Charlottesville, VA, United States; ^4^Brain Institute, University of Virginia, Charlottesville, VA, United States; ^5^Department of Neurosurgery, University of Virginia Health System, Charlottesville, VA, United States

**Keywords:** Parkinson's disease, magnetic resonance imaging, gray matter, atrophy, voxel-based morphometry, braak hypothesis

## Abstract

Models which assess the progression of Lewy pathology in Parkinson's disease have proposed ascending spread in a caudal-rostral pattern. *In-vivo* human evidence for this theory is limited, in part because there are no biomarkers that allow for direct assessment of Lewy pathology. Here, we measured neurodegeneration via MRI, an outcome which may serve as a proxy for a more direct assessment of ascending models using a combination of (1) MRI-based measures of gray matter density and (2) regions of interest (ROIs) corresponding to cortical and subcortical loci implicated in past MRI and stereological studies of Parkinson's disease. Gray matter density was measured using brain MRI voxel-based morphometry from three cohorts: (1) early Parkinson's disease, (2) more advanced Parkinson's disease and (3) healthy controls. Early Parkinson's disease patients (*N* = 228, mean age = 61.9 years, mean disease duration = 0.6 years) were newly diagnosed by the Parkinson's Progression Markers Initiative (PPMI). Advanced Parkinson's disease patients (*N* = 136, mean age = 63.5 years, mean disease duration = 8.0 years) were collected retrospectively from a local cohort undergoing evaluation for functional neurosurgery. Control subjects (*N* = 103, mean age = 60.2 years) were from PPMI. Comparative analyses focused on gray matter regions ranging from deep gray subcortical structures to the neocortex. ROIs were defined with existing probabilistic cytoarchitectonic brain maps. For subcortical regions of the basal forebrain, amygdala, and entorhinal cortex, advanced Parkinson's disease patients had significantly lower gray matter density when compared to both early Parkinson's disease and healthy controls. No differences were seen in neocortical regions that are “higher” in any proposed ascending pattern. Across early and advanced Parkinson's disease, gray matter density from nearly all subcortical regions significantly decreased with disease duration; no neocortical regions showed this effect. These results demonstrate that atrophy in advanced Parkinson's patients compared to early patients and healthy controls is largely confined to subcortical gray matter structures. The degree of atrophy in subcortical brain regions was linked to overall disease duration, suggesting an organized pattern of atrophy across severity.

## Introduction

Parkinson's disease, a progressive neurodegenerative disorder, is characterized by cell death within the substantia nigra and presence of misfolded α-synuclein aggregates known as Lewy Bodies throughout the brain parenchyma (1–3). Braak and colleagues first described the pathological progression of α-synuclein aggregates in PD as occurring in an organized and ascending manner, beginning in subcortical brain structures and ending in the neocortex (4). Braak's model and others which propose ascending spread are underpinned by the “prion hypothesis,” which posits that α-synuclein aggregates are taken up by neurons, undergo axonal transport, and are transferred to other neurons as part of a prion-like process (5). Recently, this model has been challenged (6), and other theories have emerged which attempt to explain why large subsets of PD patients do not follow proposed ascending patterns (7).

Important elements of hypotheses proposing ascending spread in PD enjoy support from *ex-vivo* (8–10), *in-vitro* (11–13) and *in-vivo* animal (2, 14, 15) studies of Parkinson's disease. However, because there is currently no method for the detection of α-synuclein aggregates in living human patients (16), there is a general lack of human *in-vivo* support for ascending spread models. As a proxy for Lewy pathology, many studies have utilized MRI methods to study neurodegeneration in PD. Though it is important to note that evidence connecting Lewy pathology and neuronal death is limited and neurodegeneration is an imperfect proxy for Lewy pathology (16, 17), neurodegeneration may nevertheless follow an ascending pattern in PD that is measurable via MRI techniques.

Qualitative evaluation of T1- and T2-weighted “conventional” structural MRI is generally considered normal in early PD patients (18, 19). More advanced techniques, which allow for quantitative evaluation of changes to tissue structure, provide an opportunity to study neurodegeneration in PD (20). The literature employing MRI to measure PD neurodegeneration has utilized techniques such as such as voxel and deformation-based morphometry (21–24), cortical thickness (25, 26), functional MRI (27–29), and free-water diffusion (30, 31) amongst numerous other methods. However, the MRI literature as a whole has reported inconsistent findings with respect to neurodegeneration (32, 33), perhaps due to methodological issues related to the lack of a standardized protocol for measuring atrophy in brain regions. With respect to PD progression, few MRI studies have attempted to study the disease process as a whole, tending instead to focus on individual ROIs or small subsets of ROIs. Studies in notable exception to the previous statement have introduced evidence both for and against ascending spread of neurodegeneration (23, 25), underscoring the need for further investigation.

A difficulty inherent to any study of progressive PD neurodegeneration is the disparate methods required to properly measure the brain regions known to degenerate in PD. For example, the substantia nigra is difficult to quantify with conventional MRI sequences due to the disparate magnetic properties of gray matter and neuromelanin (34). Successful quantification of substantia nigra degenereation has generally relied on combinations of T1-, T2, and T2*-weighted imaging (35) or attempts to image neuromelanin directly (36, 37). The small size and complexity of brainstem regions such as the locus coeruleus require the use of ultrahigh-field MRI scanners for proper resolution (38). Due to these challenges, no single MRI method is currently appropriate for measuring the earliest regions involved in the proposed ascending pattern of neurodegeneration in PD. However, voxel-based morphometry (VBM), a technique known to agree with findings from autopsy studies and other imaging modalities (39–41), allows for the measurement of many gray matter structures implicated in PD ranging from basal forebrain to the neocortex. Past VBM studies of PD have identified neurodegeneration in the basal forebrain (21, 22), amygdala (24, 42), hippocampus (43, 44), and neocortex (42, 45).

Here, we aimed to evaluate neurodegeneration in PD patients through measurements of gray matter density (GMD) obtained through VBM processing of T1-weighted MRI. This study focused on a specific subset of gray matter ROIs which were: (1) implicated by ascending models for PD, (2) demonstrated consistent findings between past MRI and stereological PD studies, and (3) could be measured by a combination of VBM and existing brain maps. Sub-regions of the basal forebrain (22, 46–48), amygdala (24, 42, 49), hippocampus (44, 50–52), and neocortex (23, 26, 53) all met the aforementioned criteria. Whole-brain GMD was obtained from three cohorts delineated by their disease duration: (1) early Parkinson's disease patients, (2) more advanced Parkinson's disease patients, and (3) healthy control subjects. Regional GMD was extracted according to existing MRI maps and compared between the groups to test the general hypothesis that patients with more advanced Parkinson's disease would show decreases in GMD in subcortical regions with relative sparing of cortical regions.

## Materials and Methods

### Subjects

#### Healthy Controls

One hundred and three healthy control subjects (males = 67, females = 36, mean age = 60.2 years) were obtained from the PPMI database. The PPMI study is well-described at ppmi-info.org. Briefly, PPMI is a comprehensive multi-center study designed to identify biomarkers of PD with the goal of improving evaluation of disease modifying therapeutics (54). Healthy control subjects included in PPMI were above 30 years of age, had never been diagnosed with any major neurological disorder, had no first-degree relatives with idiopathic PD, and were not cognitively impaired based on a score of 26 or above on the Montreal Cognitive Assessment. Healthy controls included in this study had a 3D Magnetization Prepared Rapid Acquisition Gradient Echo (MP-RAGE) MRI sequence acquired on a Siemens MRI scanner at baseline event. Control subjects were excluded from analysis in this study if a non-MPRAGE sequence such as Spoiled Gradient Echo (SPGR) was acquired, or if a non-Siemens MRI scanner was used. Subjects were also excluded if images did not meet quality control standards described below.

#### Early Parkinson's Disease

Two hundred and twenty-eight early Parkinson's disease subjects (males = 141, females = 87, mean age = 61.9 years) were obtained from the baseline time point of the longitudinal PPMI database. The PPMI study is well-described at ppmi-info.org. Parkinson's Disease subjects included in PPMI were above 30 years of age, had received a clinical diagnosis of PD within 2 years of screening, had not taken nor were expected to take PD medication within 6 months of enrollment, had not received a clinical diagnosis of dementia, and had their PD diagnosis confirmed by DaTscan, a measurement of dopamine transporter which is known to have a high degree of specificity for PD (55). Early PD subjects included in this study had a 3D Magnetization Prepared Rapid Acquisition Gradient Echo (MP-RAGE) MRI sequence acquired on a Siemens MRI scanner at the baseline event. Early PD subjects were excluded from analysis in this study if a non-MPRAGE sequence such as Spoiled Gradient Echo (SPGR) was acquired, or if a non-Siemens MRI scanner was used. Subjects were also excluded if images did not meet quality control standards described below.

#### Advanced Parkinson's Disease

One hundred and thirty-six advanced PD subjects (males = 101, females = 35, mean age = 63.5 years) were obtained from a cohort of patients who underwent a first neurosurgical procedure to treat motor symptoms between January 1, 2010 and July 1, 2016 at the University of Virginia. Patients presenting for these procedures have generally carried the PD diagnosis for multiple years and have a motor symptom that is not (or inconsistently) responsive to oral medication. The procedures included deep brain stimulation, radiofrequency ablation, and focused ultrasound. The diagnosis of Parkinson's disease was confirmed by a movement disorders neurologist as part of the pre-surgical evaluation. Subjects with a 3D MP-RAGE MRI which met quality control standards were included for analysis in this study. Subjects were excluded if a non-MPRAGE sequence was acquired, or if imaging data was not acquired as part of the presurgical procedure. No post-surgically acquired MRI was included for analysis in this study. The University of Virginia Institutional Review Board for Health Sciences Research approved the retrospective collection of MRI images and medical information from patients for use in this study. On the basis of having carried the diagnosis longer than the early PD group, we refer to these patients as “Advanced” throughout the remainder of the manuscript.

#### MRI Acquisition and Quality Control

Healthy control and early PD MRI images were acquired with the MP-RAGE T1 weighted MRI sequence on Siemens MRI scanners. MRI sequence information can be found in the MRI technical operations manual (http://www.ppmi-info.org/wp-content/uploads/2017/06/PPMI-MRI-Operations-Manual-V7.pdf). PPMI image acquisition guidelines required slice thickness of 1.5 mm or less and no interslice gap, with repetition (TR) and echo (TE) time varying according to suggested settings at each site. PPMI images were obtained from the PPMI imaging database on 1/04/2017. Advanced PD images were also acquired with an MP-RAGE T1 sequence on Siemens MRI scanners. MRIs for the advanced PD cohort were obtained as part of routine clinical care on multiple MRI scanners, resulting in less standardization of MP-RAGE sequence parameters than found in PPMI. Voxel size in healthy control and early PD images measured either 1 × 1 × 1 mm or 1 × 1 × 1.2 mm while advanced PD voxel size varied but did not exceed 1 × 1 × 1.2.

All images analyzed in this study were processed through the automated quality control function contained within the CAT12 toolbox (http://dbm.neuro.uni-jena.de/cat/) in MATLAB. This tool considers noise, inhomogeneities, and image resolution to create a composite score on a scale of A to E. Only images receiving a composite score of B- or higher, corresponding to “good” image quality, were included for final analysis in this study.

### Voxel Based Morphometry Processing

Regional gray matter density was calculated from all images according to previously published methods (56). Processing took place in two main steps: (1) preprocessing via voxel-based morphometry and (2) application of cytoarchitectonic probabilities to processed gray matter density volumes. Voxel-based morphometry (57, 58) was applied to all images from the MIRIAD database using the CAT12 toolbox (http://www.neuro.uni-jena.de/cat/) within SPM12 (Wellcome Department of Imaging Neuroscience Group, London, UK; http://www.fil.ion.ucl.ac.uk/spm). The VBM analysis pipeline has been described at length previously (59, 60). Briefly, before processing, the origin of each image was manually reoriented to the anterior commissure in SPM12. Within the CAT12 toolbox, images were denoised according to spatial-adaptive Non-Local Means (SANLM) denoising (61) and Markov Random Field (62) approaches. Images were bias-corrected, spatially normalized to standard stereotactic space with an affine registration, and a local intensity transformation was performed. Normalized images were segmented into gray matter, white matter, and cerebrospinal fluid according to the Adaptive Maximum A Posterior (AMAP) technique (62). Lorio et al.'s (63) tissue priors were used for spatial normalization, skull-stripping, and initial segmentation estimate within the AMAP segmentation. Partial Volume Estimation (64) estimated partial volume fractions to account for voxels which may contain more than one tissue type. The Diffeomorphic Anatomic Registration Through Exponentiated Lie [DARTEL; (65)] algorithm as well as Geodesic Shooting (66) were used to register segmented images into standard MNI space. Finally, segmented images were modulated by the amount of volume changes from the spatial registration to preserve the total amount of gray matter.

### Regions of Interest

Region specific GMD was measured according to cytoarchitectonic probabilistic maps for the reference MNI single subject brain that were derived from 3D reconstruction of histological sections from post mortem brains. Regional GMD was calculated using a custom MATLAB script, which multiplied the GMD value for each voxel by the weighting contained within the probabilistic map. Weighted GMD values were summed bilaterally, and then standardized by dividing each image by the sum of the weighting contained within each probabilistic mask. All probabilistic tissue maps were obtained from the Anatomy Toolbox, contained within SPM12, and moved from the anatomical space of the single subject MNI template into standard MNI template space by an affine translation along the y and z axes of 4 and 5 mm (67).

From the Anatomy Toolbox Version 2.2b, 13 subcortical cytoarchitechtonically-defined regions of interest were selected: Ch4 of the basal forebrain [abbr: Ch4; (68)], Ch1-3 of the basal forebrain [abbr: Ch1-3; (68)], centromedial amygdala [abbr: CM; (69)], laterobasal amygdala [abbr: LB; (69)] superficial amygdala [abbr: SF; (69)], hippocampal-amygdala transition area [abbr: HATA; (69)], amygdala-striatal transition area [abbr: ASTR; (69)], entorhinal cortex [abbr: EC; (69)], CA1 of the hippocampus [abbr: CA1, (69)], CA2 of the hippocampus [abbr: CA2; (69)], CA3 of the hippocampus [abbr: CA3; (69)], subiculum [abbr: SUBC; (69)], and dentate gyrus [abbr: DG; (69)]. The Anatomy Toolbox contains a list of more than 50 possible cytoarchitectonically-defined neocortical regions to consider. To survey a similar number of cortical regions while limiting the number of statistical comparisons, we chose a group of 14 neocortical regions: primary motor cortex area 4a [abbr: PMC 4a; (70)], primary motor cortex area 4p [abbr: PMC 4p; (70)], primary auditory cortex are TE 1.0 [abbr: TE 1.0; (71)], primary auditory cortex are TE 1.1 [abbr: TE 1.1; (71)], primary auditory cortex are TE 1.2 [abbr: TE 1.2; (71)], secondary auditory cortex are TE 3 [abbr: TE 3; (71)], primary somatosensory cortex area 1 [abbr: PSC 1; (72)], primary somatosensory cortex area 2 [abbr: PSC 2; (72)], primary somatosensory cortex area 3a [abbr: PSC 3a; (72)], primary somatosensory cortex area 3b [abbr: PSC 3b; (72)], Broca's area 44 [abbr: BA 44; (73)], Broca's area 45 [abbr: BA 45; (73)], occipital cortex area V1 [abbr: V1; (74)], and occipital cortex area V2 [abbr: V2; (74)]. Table 1 lists each of the brain regions measured in this study and the reference for how that region was defined.

**Table 1:** Complete list of brain regions included for analysis.

**Region of interest**	**References**
Ch4 Basal Forebrain	(68)
Ch 1-3 Basal Forebrain	(68)
Centromedial Amygdala	(69)
Laterobasal Amygdala	(69)
Superficial Amygdala	(69)
Amygdala-Striatal Transition Area	(69)
Hippocampal-Amygdala Transition Area	(69)
Entorhinal Cortex	(69)
CA1 Hippocampus	(69)
CA2 Hippocampus	(69)
CA3 Hippocampus	(69)
Subiculum	(69)
Dentate Gyrus	(69)
4a Primary Motor Cortex	(70)
4p Primary Motor Cortex	(70)
TE 1.0 Primary Auditory Cortex	(71)
TE 1.1 Primary Auditory Cortex	(71)
TE 1.2 Primary Auditory Cortex	(71)
TE3 Secondary Auditory Cortex	(75)
1 Primary Somatosensory Cortex	(72)
2 Primary Somatosensory Cortex	(72)
3a Primary Somatosensory Cortex	(72)
3b Primary Somatosensory Cortex	(72)
V1 Occipital Cortex	(74)
V2 Occipital Cortex	(74)
44 Broca's Area	(73)
45 Broca's Area	(73)

### Statistical Methods

#### Participant Characteristics

For the early PD, advanced PD, and healthy control groups, sex distributions were summarized as frequencies and percentages and ages were summarized as mean, standard deviation (SD), and range of the empirical distribution. Sex frequencies were compared using Fisher's exact-test and ages were compared using the two-sample *Student's* t-test.

#### Gray Matter Density and Age Associations

Among all Parkinson's disease cases, ordinary least-squares (OLS) linear regression was utilized to examine, for each test-region of the brain, the association between GMD and chronological age. With regard to the OLS linear regression model specification, GMD functioned as the dependent variable and chronological age (yrs.) functioned as the independent variable. The focus of hypothesis testing was on testing if the OLS linear regression slope parameter is equal to zero with the restriction that the hypothesis testing procedure is carried out in a way in which among all 27 test-regions of the brain, no more than 5% of rejected null hypotheses will be in error. With this focus in mind, for each of the 27 test-regions of the brain, a *p*-value was calculated based on the regression slope coefficient F-statistic and the corresponding F-distribution of the F-statistic that reflected the chance of observing a F-statistic as large or larger when the null hypothesis is true: i.e., the underlying slope of the relationship between GMD and chronological age is equal to 0. The complete set of 27 *p*-values were sorted in ascending order and compared to the corresponding Benjamini & Hochberg (BH) false positive discovery error rate rejection threshold (76), which was computed under the restriction that among all null hypotheses rejected, no more than 5% of the rejected null hypotheses will be in error. For test-regions of the brain in which the *p*-value was less than the BH false positive discovery error rate threshold, the null hypothesis that the true value of the regression slope parameter is equal to zero was rejected.

#### Between-Group Differences in Gray Matter Density

Per test-region of the brain, the distributions of GMD were compared between the early PD patients, advanced PD patients, and healthy controls by way of analysis of covariance (ANCOVA). Three sets of ANCOVAs were conducted. One set of ANCOVAs was focused on comparing the GMD of the early PD patients and the advanced PD patients, while a second set of ANCOVAs was focused on comparing the GMD of the advanced PD patients and the healthy controls, and the third set of ANCOVAs was focused on comparing the GMD of early PD patients and healthy controls. All three sets of ANCOVAs were conducted using the same model specification. The dependent variable was the test-region log_e_(GMD) and the independent factor of interest was a categorical variable that distinguished the members of one study-group (e.g., advanced PD patients) from members of the other study-group (e.g., early PD patients). Chronological age and sex served as covariate adjustments in the ANCOVA model. Variables which were completely confounded between the early PD and advanced PD groups (e.g., medication status) were not included in the model. Per ANCOVA, a chronological age and sex adjusted linear contrast of least-squared means was used to test the null hypothesis that the covariate adjusted mean log_e_(GMD) is the same irrespective of the study-group. Since each set of between-group comparisons required testing a total of 27 null hypotheses (i.e., one per test-region of the brain), the BH false discovery error rate control procedure (see above) was used so that no more than 5% of rejected null hypotheses will be in error.

#### Gray Matter Density Disease Duration Association

Among all PD cases, OLS linear regression was utilized to examine, for each test-region of the brain, the association between GMD and disease duration (yrs.). The analytical methods and hypothesis testing strategy that were used to examine the associations between GMD and disease duration were identical to the analytical methods and hypothesis testing strategy that were used to examine the associations between GMD and chronological age.

#### Differences in GMD Slope Directionality

For each test-region of the brain, the distributions of OLS linear regression slope directionality were compared between cortical and subcortical brain regions (as delineated in the experimental methods) separately for early PD and advanced PD patients by Fischer's exact test. Slope directionality was based on sign of expression and not dependent on the statistical significance.

## Results

GMD measurements from, 228 early PD, 136 advanced PD, and 103 control subjects were included for analysis in this study (Table 2). With respect to sex distribution, males represented the majority of the subjects of each group. 61.8% of the early PD were male, 74.3% of the advanced PD were male, and 65.0% of the healthy controls were male, with males more highly represented in the advanced PD group than in the early PD group (*p* = 0.016). The advanced PD group was older (mean = 63.5 yrs., SD = 8.7 yrs., range = [38.8, 91.1 yrs.]) than the healthy control group (mean = 60.2 yrs., SD = 11.2 yrs., range = [31.1, 82.1 yrs.]) (*p* = 0.01), and trended older than the early PD group (mean = 61.9 yrs., SD = 9.5 yrs., range = [33.7, 82.9 yrs.]) (*p* = 0.10). The advanced PD group had longer disease duration (mean = 8.0 yrs., SD = 4.8 yrs., range = [<0.5, 27.6 yrs.]) than the early PD group (mean = 0.6 yrs., SD = 0.6 yrs., range = [<0.5, 3.0 yrs]) (*p* < 0.001). The advanced PD group had higher mean UPDRS III score (mean = 35.1, SD = 11.2 yrs, range = [9, 64]) than the early PD group (mean = 20.7, SD = 8.7, range = [4, 47]) (*p* < 0.001). The advanced PD group had lower mean Montreal Cognitive Assessment (MoCA) score (mean = 24.9, SD = 11.2, range = [11, 30]) than the early PD group (mean = 27.1, SD = 2.4, range = [17, 30]) or the healthy control group (mean = 28.3, SD = 1.2, range = [26. 30]) (*p* < 0.001).

**Table 2:** Descriptive statistics for subjects included in the present study.

	**Early PD**	**Advanced PD**	**Controls**
Demographics			
*n*	228	136	103
Female	87	35	36
Male	141	101	67
Age at MRI (years)	61.9 ± 9.5	63.5 ± 8.7	60.2 ± 11.2
Disease duration (years)	0.6 ± 0.6	8.0 ± 4.8	0
UPDRS III total	20.7 ± 8.7	35.1 ± 11.2	0.7 ± 1.3
MoCA	27.1 ± 2.4	24.9 ± 11.2	28.3 ± 1.2

### Gray Matter Density and Age Associations

Among PD patients as a whole (i.e., non-differentiated by disease stage), GMD was negatively associated with age for 22 of the 27 test-regions of interest (Supplementary Table 1). Regions in which GMD was not associated with age were primarily located in the hippocampus (CA2, HATA, subiculum, entorhinal cortex). Additionally, laterobasal amygdala GMD was not associated with age.

### Between-Group Differences in GM Density

**Figure 1:**
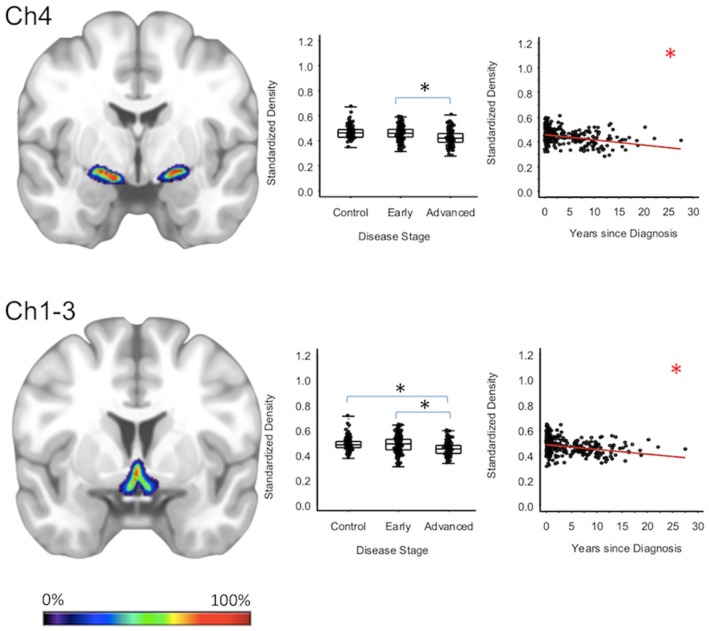
Cholinergic Basal Forebrain Regions. Group level gray matter density differences and relationship between disease duration and gray matter density. (Ch4) Ch4 nucleus of the basal forebrain. (Ch1-3) Ch1-3 nuclei of the basal forebrain. Color legend indicates probabilities contained within the mask from 0% (blue) to 100% (red). *Significant at *p* < 0.05, FDR corrected for multiple comparisons.

For subcortical regions, Ch4 GMD was 4.0% less (95% CI: [1.8, 6.1%]) for the advanced PD patients when compared to early PD patients (*p* < 0.001) and 4.3% less (95% CI: [2.0, 6.5%]) when compared to healthy controls (*p* < 0.001); while Ch4 GMD was comparable for the early PD patients and the healthy controls. Ch123 GMD was 3.7% less (95% CI: [1.4, 6.0%]) for advanced PD patients when compared to early PD patients (*p* = 0.002), but was comparable to the GM of the healthy controls. Early PD patients and healthy controls also had comparable Ch123 GMD (Figure 1, Supplementary Tables 2–4).

**Figure 2:**
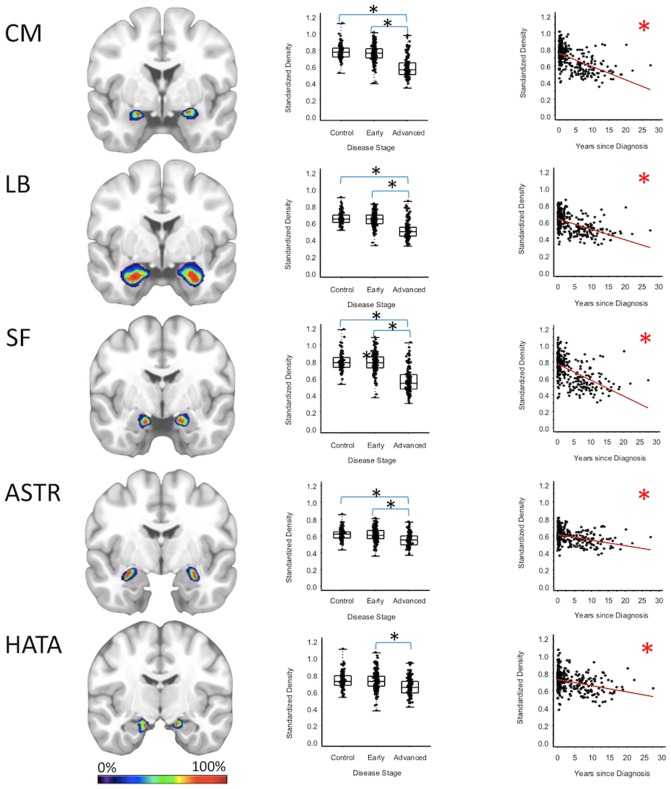
Amygdala Regions. Group level gray matter density differences and relationship between disease duration and gray matter density. (CM) Centromedial amygdala, (LB) Laterobasal amygdala, (SF) Superficial amygdala, (ASTR) Amygdala-Striatal Transition Area, (HATA) Hippocampal-Amygdala Transition Area. Color legend indicates probabilities contained within the mask from 0% (blue) to 100% (red). *Significant at *p* < 0.05, FDR corrected for multiple comparisons.

Centromedial-amygdala GMD was 15.9% less (95% CI: [13.1, 18.6%]) for the advanced PD patients when compared to the early PD patients (*p* < 0.001) and 16.5% less (95% CI: [12.3, 20.5%] when compared to the healthy controls (*p* < 0.001), while centromedial-amygdala GMD was not significantly different between the early PD patients and the healthy controls. Superficial-amygdala GMD was 19.5% less (95% CI: [14.5, 24.2%]) for the advanced PD patients when compared to early PD patients (*p* < 0.001), and 20.0% less (95% CI: [14.9, 24.7%]) when compared to the heathy controls (*p* < 0.001); while superficial-amygdala GMD was comparable for the early PD patients and healthy controls. Laterobasal-amygdala GMD was 12.6% less (95% CI: [9.1, 16.0%]) for the advanced PD patients when compared to the early PD patients (*p* < 0.001) and 13.2% less (95% CI: [9.3, 16.9%] when compared to healthy controls (*p* < 0.001); while laterobasal-amygdala GMD was comparable for the early PD patients and healthy controls. GMD of the amygdala-striatal transition area of the advanced PD patients was 6.4% less (95% CI: [3.5, 9.3%]) than the GMD of the early PD patients (*p* < 0.001) and 6.2% less (95% CI: [3.1, 9.2%]) than the GMD of healthy controls (*p* < 0.001); while amygdala-striatal transition area GMD was comparable for the early PD patients and healthy controls. HATA GMD was 7.0% less (95% CI: [3.0, 10.8%]) for the advanced PD patients when compared to the early PD patients (*p* < 0.001), and 6.9% less (95% CI: [0.80, 12.7%]) when compared to the healthy controls—which after false positive discovery adjustment was not deemed significant—while HATA GMD was comparable for the early PD patients and the healthy controls (Figure 2, Supplementary Tables 2–4).

**Figure 3:**
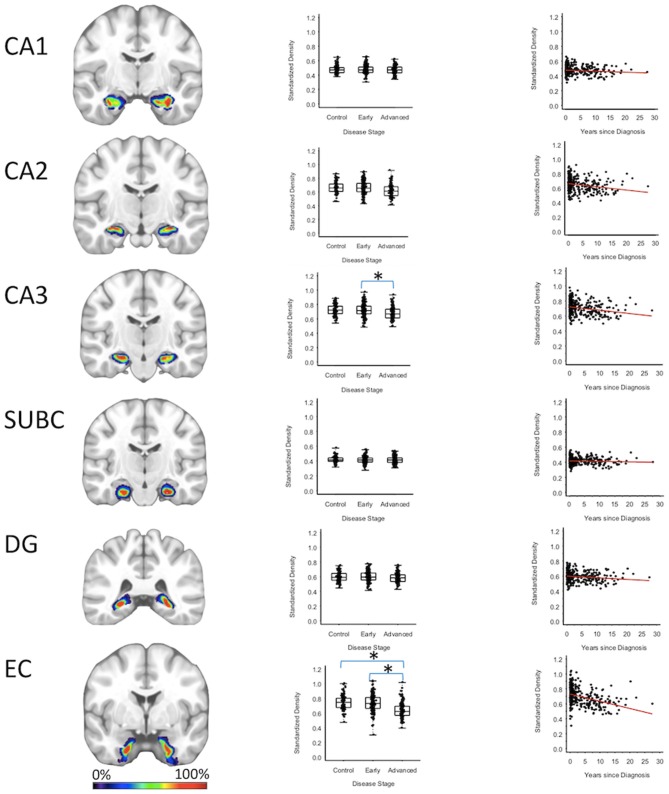
Hippocampus Regions. Group level gray matter density differences and relationship between disease duration and gray matter density. (CA1) CA1 of hippocampus, (CA2) CA2 of hippocampus, (CA3) CA3 of hippocampus, (SUBC) Subiculum, (DG) Dentate Gyrus, (EC) Entorhinal Cortex. Color legend indicates probabilities contained within the mask from 0% (blue) to 100% (red). *Significant at *p* < 0.05, FDR corrected for multiple comparisons.

Entorhinal cortex GMD was 10.1% less (95% CI: [6.0, 14.0%]) for the advanced PD patients when compared to the early PD patients (*p* < 0.001), and 10.2% less (95% CI: [5.9, 14.4%]) when compared to the healthy controls (*p* < 0.001); while entorhinal cortex GMD was comparable for the early PD patients and healthy controls. GMD of the CA3 of the hippocampus of advanced PD patients was 4.3% less (95% CI: [0.70, 7.7%]) than the GMD of the early PD patients (*p* = 0.018). The GMD of the CA3 of the hippocampus was comparable for the advanced PD patients and the healthy controls and comparable for the early PD patients and healthy controls (Figure 3, Supplementary Tables 2–4).

**Figure 4:**
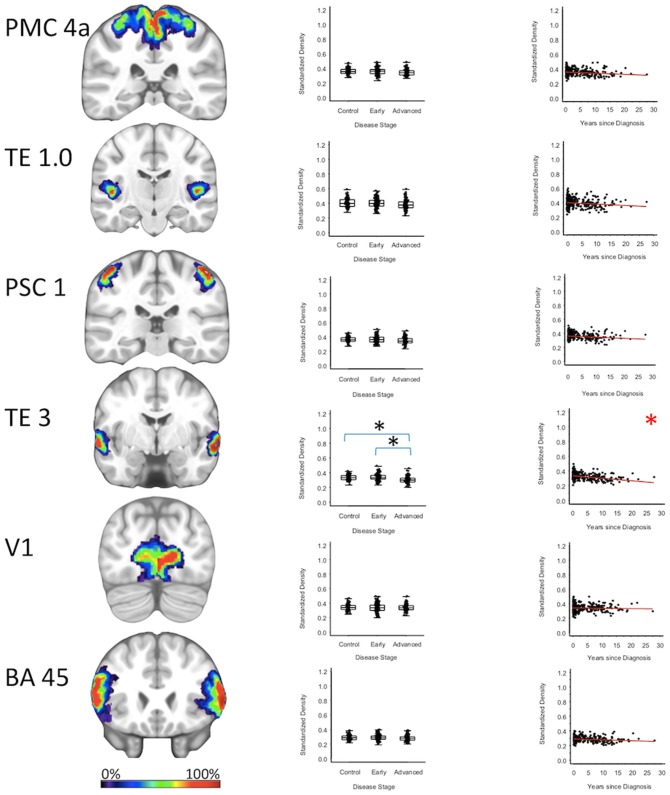
Neocortical Regions. Group level gray matter density differences and relationship between disease duration and gray matter density. (PMC4a) Primary motor cortex area 4a (TE1.0) Primary auditory area TE1.0 (PSC1) Primary somatosensory cortex area 1 (TE3) Secondary auditory area (TE3 V1) Area V1 of the occipital cortex (BA45) Broca's area defined by Brodmann area 45. Color legend indicates probabilities contained within the mask from 0% (blue) to 100% (red). *Significant at *p* < 0.05, FDR corrected for multiple comparisons.

For cortical regions, the secondary auditory area TE3 GMD was 3.5% less (95% CI: [1.8, 5.3%]) for the advanced PD patients when compared to the early PD patients (*p* < 0.001), and 3.2% less (95% CI: [1.4, 4.9%]) when compared to the healthy controls (*p* = 0.001). The secondary auditory area TE3 GMD was comparable between early PD patients and the healthy controls. Between-group comparisons of the GMDs of remaining stage six regions (i.e., the primary motor cortex, primary somatosensory cortex, occipital cortex, primary auditory cortex, and Broca's area) showed the GMD of the early PD, advanced PD, and healthy control groups to be not significantly different after false positive discovery adjustment (Figure 4, Supplementary Figures 1, 2, Supplementary Tables 2–4).

### Gray Matter Density and Disease Duration Associations

To confirm that group level differences in regional GMD were related to disease duration, a set of regression analyses that examined the relationship between GMD and disease duration controlling for chronological age and sex was performed. The individual regression analyses were conducted using the data of both early and advanced PD patients to increase the range of disease duration present in the sample. For subcortical regions, Ch4 GMD declined with disease duration (slope = −0.010 units/yr; 95% CI: [−0.015,−0.005], *p* < 0.001) and Ch123 GMD declined with disease duration (slope = −0.008 units/yr; 95% CI: [−0.013,−0.002], *p* = 0.009) (Figure 1, Supplementary Table 5). The centromedial amygdala GMD declined with disease duration (slope = −0.025 units/yr; 95% CI: [−0.033,−0.017], *p* < 0.001), superficial amygdala GMD declined with disease duration (slope = −0.031 units/yr; 95% CI: [−0.040,−0.021], *p* < 0.001), laterobasal amygdala GMD declined with disease duration (slope = −0.023 units/yr; 95% CI: [−0.040,−0.005], *p* = 0.013), amygdala-striatal transition area GMD declined with disease duration (slope = −0.012 units/yr; 95% CI: [−0.017,−0.006], *p* < 0.001), and HATA GMD declined with disease duration (slope = −0.011 units/yr; 95% CI: [−0.017,−0.004], *p* = 0.001) (Figure 2). Entorhinal cortex GMD declined with disease duration (slope = −0.014 units/yr; 95% CI: [−0.022,−0.007], *p* < 0.001) (Figure 3). For cortical regions, only the GMD of the secondary auditory association area TE3 declined with disease duration (slope = −0.011 units/yr; 95% CI: [−0.017, −0.005], *p* < 0.001) (Figure 4, Supplementary Table 5).

### Regional Differences in GMD Slope Directionality

To further test differences in disease progression between early and PD groups, a comparison of the directionality of the early and PD slope parameters for the age and sex adjusted relationships between duration of disease and GMD was performed. For subcortical regions in early PD, one region (7.7%) had a positive slope, compared to 12 regions (92.3%) which had a negative slope. For subcortical regions in advanced PD patients, all 13 regions (100%) had a negative slope. For cortical regions in early PD, 13 regions (92.9%) had a positive slope, compared to one region (7.1%) which had a negative slope. For cortical regions in advanced PD patients, all 14 regions (100%) had a negative slope. The difference in the distribution of positive and negative slopes for subcortical and cortical regions in early PD is significant (*p* < 0.001). Additionally, the difference in the distribution of positive and negative slopes for cortical regions between early PD and advanced PD is significant (*p* < 0.001).

## Discussion

Loss of gray matter in Parkinson's disease is pathological, and additive to the normal aging process (45, 77, 78). This study found pathological gray matter degeneration in subcortical regions of the cholinergic basal forebrain and amygdala, but found general sparing of hippocampal and neo-cortical regions in a population of advanced Parkinson's disease patients. These findings suggest a pattern that could be described roughly as “ascending” when comparing groups differentiated by disease duration.

MRI study of Parkinson's disease is extensive, but results have not demonstrated a consistent pattern of disease related atrophy (33). Previous studies have identified gray matter atrophy in early non-demented Parkinson's disease subjects (79, 80), while others have found no differences when compared to healthy controls (81–83). In advanced Parkinson's disease subjects, studies have found gray matter atrophy in the hippocampus (44, 52), amygdala (44, 84), and caudate (85). The advanced Parkinson's disease group evaluated in this study showed extensive degeneration of the basal forebrain and amygdala when compared to healthy control and early Parkinson's disease groups, but showed limited degeneration in the hippocampus and neocortex. When compared to previous neuroimaging studies, results from this study place the advanced Parkinson's disease group at the threshold of advanced disease, where degeneration in meso- or neo-cortical areas has either not occurred, or not yet resulted in measurable loss of GMD.

This study did not identify regional differences in GMD between early Parkinson's disease and healthy control groups. These findings fit within a literature that has failed to consistently detect atrophy in MRI studies of early Parkinson's disease (86, 87). A notable exception to past negative findings are deformation based morphometry studies of PPMI Parkinson's disease subjects at baseline which identified atrophy in subcortical regions such as the basal ganglia, basal forebrain, and hippocampus and predicted pathological spread of PD to subcortical regions ahead of cortical regions (23, 25). However, Zeighami et al. also identified extensive cortical atrophy, a finding not expected from a sample acquired at baseline of motor diagnosis by PPMI (23). To address the discrepancy between these studies, we compared the directionality of the early and advanced Parkinson's disease slope parameters for relationships between disease duration and regional GMD. For early Parkinson's disease subjects, 92.3% of subcortical regions displayed the expected negative slope, compared to only 7.1% of cortical regions. For advanced Parkinson's disease patients, 100% of cortical and subcortical regions displayed a negative slope. This ascending pattern of isolated subcortical atrophy in early PD patients which did not reach the level of traditional statistical significance may result from the nature of gray matter density as a downstream proxy for potential mechanisms of neurodegeneration in Parkinson's disease. Perhaps a lag exists between still unknown neurodegenerative mechanisms and ability to detect loss of gray matter density via VBM. MRI methods capable of measuring tissue microstructure (e.g., diffusion MRI) or physiology (e.g., functional MRI) may be better equipped to detect differences which precede those detectable by VBM.

A novel finding of this study is the consistency with which each region of the amygdala was shown to have degenerated in the advanced Parkinson's disease group. The amygdala, which is extensively connected to the cortex, diencephalon, basal ganglia, and olfactory bulb is associated with a number of functions in humans including learning and memory, emotion regulation, and motivation (69, 88). Degeneration of the amygdala in Parkinson's disease and other neurodegenerative diseases such as AD and dementia with Lewy bodies may result in common behavioral and cognitive deficits (88). Specifically in Parkinson's disease, the amygdala is known to undergo severe pathological change over time (89). Pathological study of the amygdala in advanced Parkinson's disease subjects has identified a selective pattern of degeneration localized to the corticomedial and basolateral formation and an association between pathological load in the laterobasal amygdala and visual hallucinations (49). MRI studies of the amygdala in Parkinson's disease have contributed mixed findings to date; reporting no difference between Parkinson's disease and control subjects (90) or slightly reduced volume of the right amygdala in Parkinson's disease relative to healthy controls (91, 92). The current study reports significant atrophy in advanced Parkinson's disease relative to early Parkinson's disease and healthy control subjects, a finding which has not previously been reported using MRI methodology. Evidence from the current study most strongly supports prior histological evaluation that showed significant regional amygdala pathology (49), perhaps due to our use of histologically defined brain maps.

Prior MRI studies of Parkinson's disease, which have not consistently identified the amygdala as an area of degeneration in Parkinson's disease, differ from the current study through application of deterministic mapping, which allows each voxel in a brain region to contribute equally to an output measure (93, 94). This study utilized probabilistic mapping, which allows voxels to differentially contribute to an output measure and account for the likelihood that they accurately reflect anatomical boundaries in an individual (69, 95). The use of histologically defined probabilistic maps is particularly indicated in the case of brain regions, such as the amygdala, which can only be properly delineated by cytoarchitecture, and may have poor or limited contrast in structural MRI (69). The consistent degeneration identified in the amygdala in this study possibly reflects this methodological difference with prior studies. These findings implicate the amygdala as an area of future MRI investigation in Parkinson's disease.

Voxel based morphometry is sensitive to effects of age (96), which manifest as wide-ranging reductions in cortical and subcortical gray matter over time (97, 98). This study attempted to control for these effects by selecting cohorts with similar mean age, but which differed by PD disease duration. Though considered a strength of this study, this design choice may limit our ability to make inferences about the “typical” pattern of atrophy in advanced PD. Recent study of outcomes in PD identified age at PD diagnosis as a strong predictor of progression to disease milestones and death (99). Older age at PD onset is associated with more severe motor and non-motor phenotypes and greater dopaminergic impairment (100). Because the advanced PD group in this study was diagnosed on average 5.8 years earlier than the early PD group, it is possible that they belong to a PD subtype which progressed to reductions in subcortical GMD at a different rate than the early PD group, and that these groups may have different relationships between GMD and duration of disease.

The major limitation of this study design stems from retrospective collection of data in the advanced Parkinson's disease group. The acquisition of the neuroimaging and neuropsychological data from the advanced PD patients took place as part of routine clinical care, and not as part of a standardized protocol. As a result, MRI images from advanced PD patients had more variable acquisition parameters and were acquired on multiple different Siemens MRI scanners. Conversely, PPMI images were acquired with relatively standardized acquisition parameters on fewer MRI scanners from different vendors. Unfortunately, there are no consensus methods for retrospective data harmonization of brain MRI structural data, a problem that was noted in a recent survey of the neuroimaging community (101, 102). Due to a noted vulnerability of datasets to cross-vendor harmonization issues (101, 102), we also chose to exclude all PPMI data not acquired Siemens scanners with an MP-RAGE protocol. Several elements of our data processing methods attempted to improve consistency between the groups (e.g., inspection for missing data, a robust, automated quality control protocol, consistent application of image registration techniques, and normalization steps intrinsic to the VBM method). Despite these steps, the possibility of confounds related to difference in MRI must be acknowledged; however, two important control findings provide a measure of confidence in the major findings of this study. First, negative findings between the advanced Parkinson's disease group and both PPMI groups for the majority of hippocampal and neocortical regions suggest that any potential confounds attributable to differences in MRI acquisition would need to be confined to specific *subcortical* regions. Second, the analysis examining early vs. advanced differences in regional slope directionality related to duration of Parkinson's disease showed change of slope direction in nearly all of the cortical regions; here, any potential confounds would need to be confined to a different set of *neocortical* regions. So, while it is possible that MRI acquisition differences alone could account for the specific, ascending pattern of regional atrophy identified in this study, such a finding would be highly improbable.

The current study provided *in-vivo* human evidence suggesting a pattern of neurodegeneration in Parkinson's disease in subcortical regions ahead of cortical regions. Widespread subcortical degeneration in the basal forebrain and amygdala of the advanced Parkinson's disease group compared to early Parkinson's disease or healthy controls suggests an organized degenerative process which effects subcortical structures in advance of cortical structures. Ongoing research efforts will utilize new MRI techniques and longitudinal data to further evaluate the progression of pathology during the course of Parkinson's disease.

## Data Availability Statement

All code used in calculation of GMD is available at https://github.com/DruzgalLabUVA/Ascending_Spread_MRI. MRI images and demographic information from PPMI are available to qualifying researchers following an application process. Collection of the advanced Parkinson's disease data was retrospective and release of individual MRI and demographic data is precluded by HIPAA.

## Ethics Statement

The University of Virginia Institutional Review Board for Health Sciences Research approved this study.

## Author Contributions

JB, MB, SS, WE, and TD contributed to study concept and design. JB, JF, and MB contributed to acquisition of data. JP conducted statistical analysis. JB, MB, JP, and TD contributed to analysis and interpretation of data. JB, JP, and TD drafted the manuscript. MB, JF, SS, and WE contributed to critical revision of manuscript for intellectual content.

### Conflict of Interest

The authors declare that the research was conducted in the absence of any commercial or financial relationships that could be construed as a potential conflict of interest.
