# Supplementary material for: Brain MRI Reveals Ascending Atrophy in Parkinson's Disease Across Severity
Source: Front Neurol. 2019 Dec 18;10:1329. doi: 10.3389/fneur.2019.01329 (PMC6930693; doi:10.3389/fneur.2019.01329)
Supplement: Supplementary file 1 [file Table_1.docx]

Supplementary Material

## Supplementary Figures


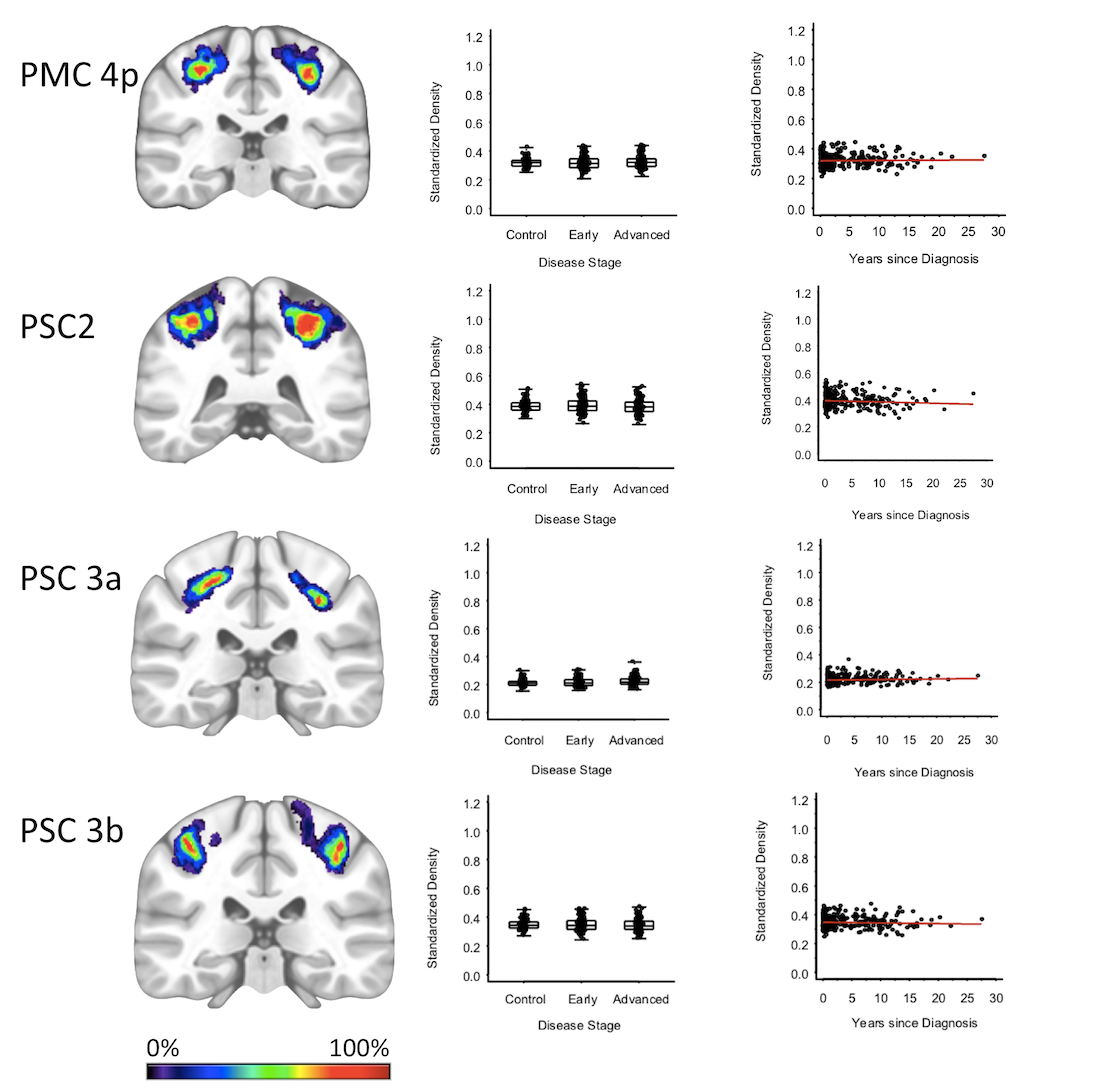


**Supplementary Figure 1.** Neocortical Regions. Group level grey matter density differences and relationship between disease duration and grey matter density. PMC4p) Primary motor cortex area 4p PSC2) Primary somatosensory cortex area 2 PSC3a) Primary somatosensory.


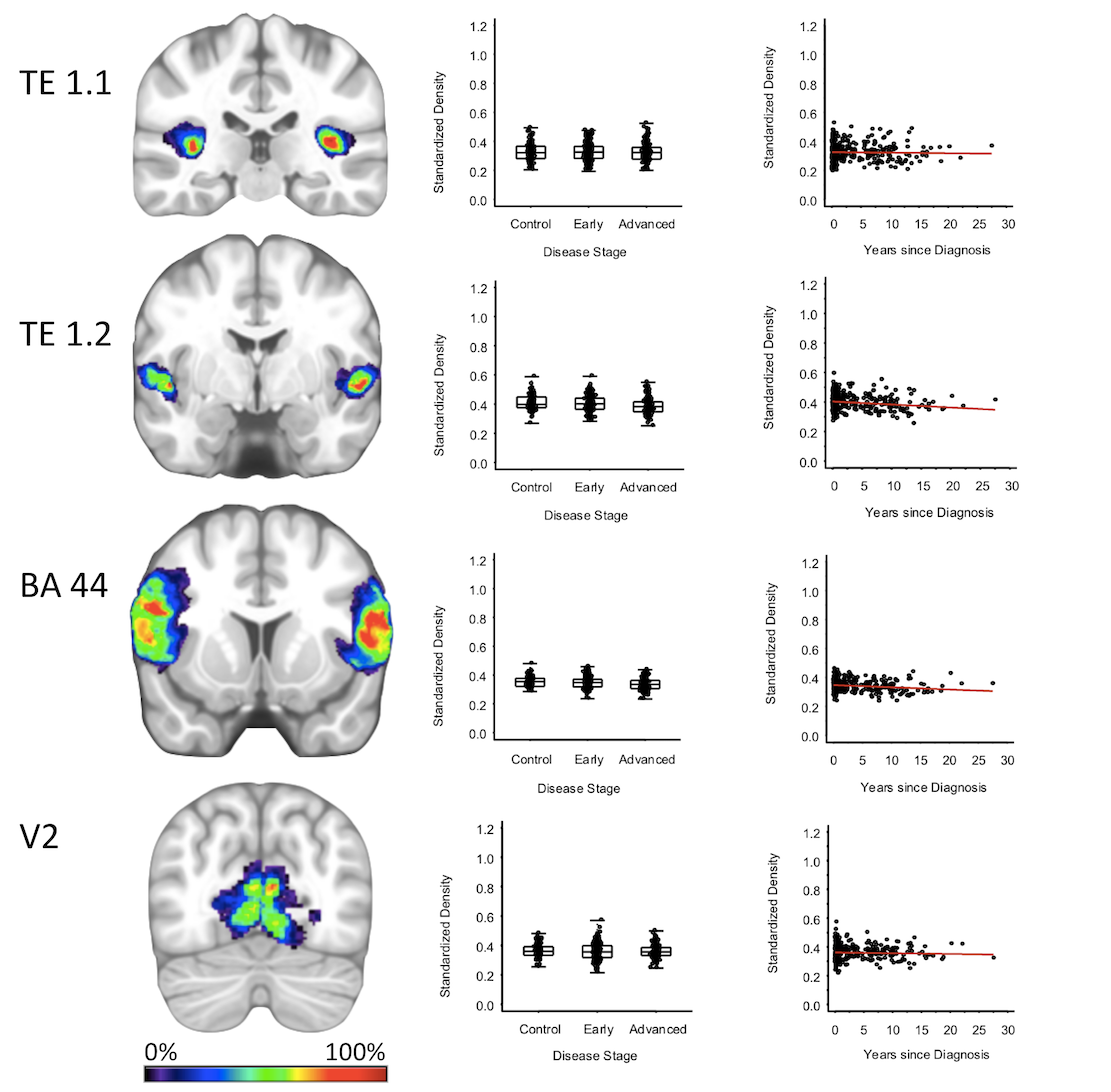


**Supplementary Figure 2.** Neocortical Regions. Group level grey matter density differences and relationship between disease duration and grey matter density. TE 1.1) Primary auditor area TE 1.1 TE1.2) Primary auditory area TE1.2 BA 44) Brodmann area 44 V1) Area V2 of the occipital cortex. Color legend indicates probabilities contained within the mask from 0%(blue) to 100%(red).

| Brain Region | Slope  (units/year) | Lower 95%  CL | Upper 95%  CL | Observed  P-value | Benjamini & Hochberg Threshold | Rejection  (Yes or No) |
| --- | --- | --- | --- | --- | --- | --- |
| Ch4 | -0.0024 | -0.0030 | -0.0018 | <0.001 | 0.00185 | Yes |
| Ch123 | -0.0030 | -0.0037 | -0.0023 | <0.001 | 0.00370 | Yes |
| Amygdala Astr | -0.0032 | -0.0042 | -0.0023 | <0.001 | 0.00556 | Yes |
| Amygdala CM | -0.0047 | -0.0062 | -0.0032 | <0.001 | 0.00741 | Yes |
| Amygdala SF | -0.0051 | -0.0070 | -0.0033 | <0.001 | 0.00926 | Yes |
| Hippocampus CA1 | -0.0023 | -0.0029 | -0.0017 | <0.001 | 0.01111 | Yes |
| Hippocampus CA3 | -0.0039 | -0.0048 | -0.0029 | <0.001 | 0.01296 | Yes |
| Hippocampus DG | -0.0030 | -0.0037 | -0.0023 | <0.001 | 0.01481 | Yes |
| Motor 4A | -0.0017 | -0.0022 | -0.0012 | <0.001 | 0.01667 | Yes |
| Motor 4P | -0.0023 | -0.0027 | -0.0018 | <0.001 | 0.01852 | Yes |
| PSC 1 | -0.0016 | -0.0021 | -0.0011 | <0.001 | 0.02037 | Yes |
| PSC 2 | -0.0021 | -0.0027 | -0.0015 | <0.001 | 0.02222 | Yes |
| PSC 3a | -0.0013 | -0.0017 | -0.0010 | <0.001 | 0.02407 | Yes |
| PSC 3b | -0.0019 | -0.0023 | -0.0014 | <0.001 | 0.02593 | Yes |
| hOC1 | -0.0018 | -0.0024 | -0.0012 | <0.001 | 0.02778 | Yes |
| hOC2 | -0.0020 | -0.0026 | -0.0014 | <0.001 | 0.02963 | Yes |
| Auditory Te1 0 | -0.0025 | -0.0032 | -0.0018 | <0.001 | 0.03148 | Yes |
| Auditory Te1 1 | -0.0021 | -0.0029 | -0.0014 | <0.001 | 0.03333 | Yes |
| Auditory Te1 2 | -0.0024 | -0.0030 | -0.0018 | <0.001 | 0.03519 | Yes |
| Auditory Te3 | -0.0014 | -0.0019 | -0.0009 | <0.001 | 0.03704 | Yes |
| Broca BA44 | -0.0018 | -0.0023 | -0.0014 | <0.001 | 0.03889 | Yes |
| Broca BA45 | -0.0013 | -0.0017 | -0.0009 | <0.001 | 0.04074 | Yes |
| Hippocampus CA2 | -0.0044 | -0.0395 | 0.0308 | 0.807 | 0.04259 | No |
| Hippocampus HATA | -0.0038 | -0.0457 | 0.0380 | 0.857 | 0.04444 | No |
| Hippocampus Subc | -0.0019 | -0.0290 | 0.0252 | 0.891 | 0.04630 | No |
| Hippocampus EC | -0.0040 | -0.0834 | 0.0753 | 0.920 | 0.04815 | No |
| Amygdala LB | -0.0034 | -0.0789 | 0.0721 | 0.929 | 0.05000 | No |

**Supplementary Table 1**. Slope parameter estimates for the regression of age at MRI onto grey matter density for Parkinson’s disease patients

| Brain Region | Ratio of Geometric Means  (Early PD : Control) | | | % Reduction  (Early PD : Control) | | | | Benjamini & Hochberg  False Discovery Criterion | | Adjustment Covariates | |
| --- | --- | --- | --- | --- | --- | --- | --- | --- | --- | --- | --- |
|  | Ratio  GM | Lower 95% CL | Upper 95% CL | % Reduction | Lower  95% CL | Upper 95%  CL | P-value | Threshold | Reject | Sex | Age MRI |
| PSC 2 | 1.011 | 0.993 | 1.029 | -1.10 | -2.90 | 0.70 | 0.242 | 0.00185 | No | 0.036 | <0.001 |
| Hippocampus DG | 1.009 | 0.986 | 1.034 | -0.90 | -3.40 | 1.40 | 0.434 | 0.00370 | No | 0.002 | <0.001 |
| PSC 1 | 1.006 | 0.990 | 1.022 | -0.60 | -2.20 | 1.00 | 0.457 | 0.00556 | No | 0.001 | <0.001 |
| PSC 3a | 1.004 | 0.993 | 1.014 | -0.40 | -1.40 | 0.70 | 0.473 | 0.00741 | No | 0.554 | <0.001 |
| Hippocampus CA3 | 1.010 | 0.981 | 1.039 | -1.00 | -3.90 | 1.90 | 0.499 | 0.00926 | No | 0.001 | <0.001 |
| Broca BA45 | 1.004 | 0.991 | 1.018 | -0.40 | -1.80 | 0.90 | 0.505 | 0.01111 | No | 0.001 | <0.001 |
| Ch123 | 1.007 | 0.987 | 1.027 | -0.70 | -2.70 | 1.30 | 0.506 | 0.01296 | No | <0.001 | <0.001 |
| PSC 3b | 1.005 | 0.990 | 1.020 | -0.50 | -2.00 | 1.00 | 0.519 | 0.01481 | No | 0.059 | <0.001 |
| Auditory Te3 | 1.005 | 0.990 | 1.019 | -0.50 | -1.90 | 1.00 | 0.520 | 0.01667 | No | <0.001 | <0.001 |
| Hippocampus CA2 | 1.009 | 0.978 | 1.040 | -0.90 | -4.00 | 2.20 | 0.592 | 0.01852 | No | 0.006 | <0.001 |
| Hippocampus CA1 | 1.005 | 0.985 | 1.025 | -0.50 | -2.50 | 1.50 | 0.645 | 0.02037 | No | <0.001 | <0.001 |
| Motor 4P | 1.003 | 0.989 | 1.017 | -0.30 | -1.70 | 1.10 | 0.677 | 0.02222 | No | 0.088 | <0.001 |
| Ch4 | 0.997 | 0.979 | 1.015 | 0.30 | -1.50 | 2.10 | 0.719 | 0.02407 | No | <0.001 | <0.001 |
| Amygdala Astr | 1.004 | 0.980 | 1.028 | -0.40 | -2.80 | 2.00 | 0.766 | 0.02593 | No | <0.001 | <0.001 |
| Amygdala CM | 0.996 | 0.966 | 1.027 | 0.40 | -2.70 | 3.40 | 0.797 | 0.02778 | No | <0.001 | <0.001 |
| hOC2 | 1.002 | 0.984 | 1.021 | -0.20 | -2.10 | 1.60 | 0.816 | 0.02963 | No | <0.001 | <0.001 |
| Hippocampus HATA | 1.004 | 0.970 | 1.038 | -0.40 | -3.80 | 3.00 | 0.830 | 0.03148 | No | <0.001 | <0.001 |
| Motor 4A | 1.001 | 0.986 | 1.017 | -0.10 | -1.70 | 1.40 | 0.856 | 0.03333 | No | <0.001 | <0.001 |
| Hippocampus EC | 1.003 | 0.967 | 1.040 | -0.30 | -4.00 | 3.30 | 0.880 | 0.03519 | No | <0.001 | <0.001 |
| Amygdala LB | 0.997 | 0.957 | 1.039 | 0.30 | -3.90 | 4.30 | 0.882 | 0.03704 | No | 0.001 | <0.001 |
| Auditory Te1 0 | 1.001 | 0.980 | 1.024 | -0.10 | -2.40 | 2.00 | 0.893 | 0.03889 | No | <0.001 | <0.001 |
| hOC1 | 1.001 | 0.983 | 1.020 | -0.10 | -2.00 | 1.70 | 0.901 | 0.04074 | No | <0.001 | <0.001 |
| Auditory Te1 1 | 1.001 | 0.978 | 1.024 | -0.10 | -2.40 | 2.20 | 0.926 | 0.04259 | No | 0.001 | <0.001 |
| Broca BA44 | 1.000 | 0.986 | 1.014 | 0.00 | -1.40 | 1.40 | 0.971 | 0.04444 | No | 0.001 | <0.001 |
| Amygdala SF | 0.999 | 0.963 | 1.037 | 0.10 | -3.70 | 3.70 | 0.971 | 0.04630 | No | <0.001 | <0.001 |
| Hippocampus Subc | 1.000 | 0.985 | 1.015 | 0.00 | -1.50 | 1.50 | 0.980 | 0.04815 | No | <0.001 | <0.001 |
| Auditory Te1 2 | 1.000 | 0.981 | 1.019 | 0.00 | -1.90 | 1.90 | 0.991 | 0.05000 | No | <0.001 | <0.001 |

**Supplementary Table 2**. Gender and age adjusted grey matter density distribution comparisons between early stage Parkinson’s disease and controls; expressed as the ratio of geometric means.

| Brain Region | Ratio of Geometric Means  (Advanced PD : Control) | | | % Reduction  (Advanced PD : Control) | | | | Benjamini & Hochberg  False Discovery Criterion | | Adjustment Covariates | |
| --- | --- | --- | --- | --- | --- | --- | --- | --- | --- | --- | --- |
|  | Ratio  GM | Lower 95% CL | Upper 95% CL | % Reduction | Lower  95% CL | Upper 95%  CL | P-value | Threshold | Reject | Sex | Age MRI |
| Amygdala SF | 0.800 | 0.753 | 0.851 | 20.00 | 14.90 | 24.70 | <0.001 | 0.002 | Yes | <0.001 | <0.001 |
| Amygdala CM | 0.835 | 0.795 | 0.877 | 16.50 | 12.30 | 20.50 | <0.001 | 0.004 | Yes | <0.001 | <0.001 |
| Amygdala LB | 0.868 | 0.831 | 0.907 | 13.20 | 9.30 | 16.90 | <0.001 | 0.006 | Yes | <0.001 | <0.001 |
| Hippocampus EC | 0.898 | 0.856 | 0.941 | 10.20 | 5.90 | 14.40 | <0.001 | 0.007 | Yes | <0.001 | <0.001 |
| Amygdala Astr | 0.938 | 0.908 | 0.969 | 6.20 | 3.10 | 9.20 | <0.001 | 0.009 | Yes | <0.001 | <0.001 |
| Ch4 | 0.957 | 0.935 | 0.980 | 4.30 | 2.00 | 6.50 | <0.001 | 0.011 | Yes | <0.001 | <0.001 |
| Auditory Te3 | 0.968 | 0.951 | 0.986 | 3.20 | 1.40 | 4.90 | 0.001 | 0.013 | Yes | <0.001 | <0.001 |
| Hippocampus HATA | 0.931 | 0.873 | 0.992 | 6.90 | 0.80 | 12.70 | 0.028 | 0.015 | No | 0.002 | 0.033 |
| Ch123 | 0.967 | 0.938 | 0.997 | 3.30 | 0.30 | 6.20 | 0.033 | 0.017 | No | <0.001 | <0.001 |
| Hippocampus CA2 | 0.961 | 0.922 | 1.001 | 3.90 | -0.10 | 7.80 | 0.053 | 0.019 | No | 0.006 | <0.001 |
| Hippocampus CA3 | 0.965 | 0.929 | 1.002 | 3.50 | -0.20 | 7.10 | 0.062 | 0.020 | No | 0.002 | <0.001 |
| PSC 3a | 1.013 | 0.999 | 1.027 | -1.30 | -2.70 | 0.10 | 0.072 | 0.022 | No | 0.077 | <0.001 |
| Auditory Te1 2 | 0.981 | 0.957 | 1.005 | 1.90 | -0.50 | 4.30 | 0.115 | 0.024 | No | 0.001 | <0.001 |
| Broca BA44 | 0.986 | 0.968 | 1.005 | 1.40 | -0.50 | 3.20 | 0.147 | 0.026 | No | 0.004 | <0.001 |
| PSC 1 | 0.986 | 0.966 | 1.006 | 1.40 | -0.60 | 3.40 | 0.172 | 0.028 | No | 0.001 | <0.001 |
| Auditory Te1 0 | 0.982 | 0.954 | 1.010 | 1.80 | -1.00 | 4.60 | 0.196 | 0.030 | No | 0.001 | <0.001 |
| Motor 4A | 0.987 | 0.968 | 1.007 | 1.30 | -0.70 | 3.20 | 0.201 | 0.031 | No | <0.001 | <0.001 |
| Motor 4P | 1.011 | 0.994 | 1.029 | -1.10 | -2.90 | 0.60 | 0.212 | 0.033 | No | 0.012 | <0.001 |
| Broca BA45 | 0.994 | 0.976 | 1.012 | 0.60 | -1.20 | 2.40 | 0.495 | 0.035 | No | 0.003 | <0.001 |
| Hippocampus DG | 0.990 | 0.961 | 1.020 | 1.00 | -2.00 | 3.90 | 0.500 | 0.037 | No | 0.002 | <0.001 |
| Hippocampus CA1 | 0.996 | 0.971 | 1.022 | 0.40 | -2.20 | 2.90 | 0.773 | 0.039 | No | <0.001 | <0.001 |
| Hippocampus Subc | 0.997 | 0.977 | 1.018 | 0.30 | -1.80 | 2.30 | 0.782 | 0.041 | No | <0.001 | <0.001 |
| PSC 3b | 1.002 | 0.983 | 1.022 | -0.20 | -2.20 | 1.70 | 0.829 | 0.043 | No | 0.002 | <0.001 |
| hOC2 | 0.998 | 0.978 | 1.018 | 0.20 | -1.80 | 2.20 | 0.831 | 0.044 | No | <0.001 | <0.001 |
| PSC 2 | 1.002 | 0.978 | 1.026 | -0.20 | -2.60 | 2.20 | 0.873 | 0.046 | No | 0.035 | <0.001 |
| Auditory Te1 1 | 0.998 | 0.969 | 1.028 | 0.20 | -2.80 | 3.10 | 0.904 | 0.048 | No | <0.001 | <0.001 |
| hOC1 | 1.000 | 0.980 | 1.019 | 0.00 | -1.90 | 2.00 | 0.961 | 0.050 | No | <0.001 | <0.001 |

**Supplementary Table 3**. Gender and age adjusted grey matter density distribution comparisons between advanced stage Parkinson’s disease and controls; expressed as the ratio of geometric means.

| Brain Region | Ratio of Geometric Means  (Advance PD : Early PD) | | | % Reduction  (Advanced PD : Early PD) | | | | Benjamini & Hochberg  False Discovery Criterion | | Adjustment Covariates | |
| --- | --- | --- | --- | --- | --- | --- | --- | --- | --- | --- | --- |
|  | Ratio  GM | Lower 95% CL | Upper 95% CL | % Reduction | Lower  95% CL | Upper 95%  CL | P-value | Threshold | Reject | Sex | Age MRI |
| Amygdala CM | 0.841 | 0.814 | 0.869 | 15.90 | 13.10 | 18.60 | <0.001 | 0.00185 | Yes | <0.001 | <0.001 |
| Amygdala SF | 0.805 | 0.758 | 0.855 | 19.50 | 14.50 | 24.20 | <0.001 | 0.00370 | Yes | <0.001 | <0.001 |
| Amygdala LB | 0.874 | 0.840 | 0.909 | 12.60 | 9.10 | 16.00 | <0.001 | 0.00556 | Yes | <0.001 | <0.001 |
| Hippocampus EC | 0.899 | 0.860 | 0.940 | 10.10 | 6.00 | 14.00 | <0.001 | 0.00741 | Yes | <0.001 | <0.001 |
| Amygdala Astr | 0.936 | 0.907 | 0.965 | 6.40 | 3.50 | 9.30 | <0.001 | 0.00926 | Yes | <0.001 | <0.001 |
| Auditory Te3 | 0.965 | 0.947 | 0.982 | 3.50 | 1.80 | 5.30 | <0.001 | 0.01111 | Yes | <0.001 | <0.001 |
| Ch4 | 0.960 | 0.939 | 0.982 | 4.00 | 1.80 | 6.10 | <0.001 | 0.01296 | Yes | <0.001 | <0.001 |
| Hippocampus HATA | 0.930 | 0.892 | 0.970 | 7.00 | 3.00 | 10.80 | 0.001 | 0.01481 | Yes | <0.001 | <0.001 |
| Ch123 | 0.963 | 0.940 | 0.986 | 3.70 | 1.40 | 6.00 | 0.002 | 0.01667 | Yes | <0.001 | <0.001 |
| Hippocampus CA3 | 0.957 | 0.923 | 0.993 | 4.30 | 0.70 | 7.70 | 0.018 | 0.01852 | Yes | <0.001 | <0.001 |
| Hippocampus CA2 | 0.955 | 0.919 | 0.993 | 4.50 | 0.70 | 8.10 | 0.022 | 0.02037 | No | 0.001 | <0.001 |
| PSC 1 | 0.981 | 0.961 | 1.000 | 1.90 | 0.00 | 3.90 | 0.054 | 0.02222 | No | <0.001 | <0.001 |
| Auditory Te1 2 | 0.981 | 0.959 | 1.005 | 1.90 | -0.50 | 4.10 | 0.117 | 0.02407 | No | <0.001 | <0.001 |
| PSC 3a | 1.010 | 0.997 | 1.024 | -1.00 | -2.40 | 0.30 | 0.134 | 0.02593 | No | 0.130 | <0.001 |
| Broca BA44 | 0.987 | 0.970 | 1.005 | 1.30 | -0.50 | 3.00 | 0.144 | 0.02778 | No | 0.001 | <0.001 |
| Auditory Te1 0 | 0.981 | 0.955 | 1.008 | 1.90 | -0.80 | 4.50 | 0.172 | 0.02963 | No | <0.001 | <0.001 |
| Motor 4A | 0.987 | 0.968 | 1.006 | 1.30 | -0.60 | 3.20 | 0.179 | 0.03148 | No | <0.001 | <0.001 |
| Hippocampus DG | 0.982 | 0.955 | 1.010 | 1.80 | -1.00 | 4.50 | 0.204 | 0.03333 | No | <0.001 | <0.001 |
| Broca BA45 | 0.990 | 0.974 | 1.006 | 1.00 | -0.60 | 2.60 | 0.233 | 0.03519 | No | 0.001 | <0.001 |
| Motor 4P | 1.010 | 0.992 | 1.028 | -1.00 | -2.80 | 0.80 | 0.292 | 0.03704 | No | 0.016 | <0.001 |
| PSC 2 | 0.992 | 0.970 | 1.015 | 0.80 | -1.50 | 3.00 | 0.485 | 0.03889 | No | 0.003 | <0.001 |
| hOC2 | 0.997 | 0.976 | 1.018 | 0.30 | -1.80 | 2.40 | 0.760 | 0.04074 | No | <0.001 | <0.001 |
| Hippocampus CA1 | 0.993 | 0.945 | 1.044 | 0.70 | -4.40 | 5.50 | 0.783 | 0.04259 | No | <0.001 | <0.001 |
| PSC 3b | 0.998 | 0.980 | 1.017 | 0.20 | -1.70 | 2.00 | 0.853 | 0.04444 | No | 0.006 | <0.001 |
| Hippocampus Subc | 0.999 | 0.981 | 1.017 | 0.10 | -1.70 | 1.90 | 0.888 | 0.04630 | No | <0.001 | <0.001 |
| Auditory Te1 1 | 0.998 | 0.971 | 1.026 | 0.20 | -2.60 | 2.90 | 0.891 | 0.04815 | No | <0.001 | <0.001 |
| hOC1 | 0.999 | 0.979 | 1.020 | 0.10 | -2.00 | 2.10 | 0.944 | 0.05000 | No | <0.001 | <0.001 |

**Supplementary Table 4**. Gender and age adjusted grey matter density distribution comparisons between advanced Parkinson’s disease and early Parkinson’s disease expressed as the ratio of geometric means.

| Brain Region | Duration of Disease | | | | Benjamini & Hochberg  False Discovery Criterion | | Adjustment Covariates | |
| --- | --- | --- | --- | --- | --- | --- | --- | --- |
|  | Slope  (units/year) | Lower 95%  CL | Upper 95%  CL | P-value | Threshold | Reject | Sex  P-value | Age  P-value |
| Amygdala SF | -0.031 | -0.040 | -0.021 | <0.001 | 0.00185 | Yes | <0.001 | <0.001 |
| Amygdala CM | -0.025 | -0.033 | -0.017 | <0.001 | 0.00370 | Yes | <0.001 | <0.001 |
| Amygdala Astr | -0.012 | -0.017 | -0.006 | <0.001 | 0.00556 | Yes | <0.001 | <0.001 |
| Hippocampus EC | -0.014 | -0.022 | -0.007 | <0.001 | 0.00741 | Yes | <0.001 | <0.001 |
| Auditory Te3 | -0.011 | -0.017 | -0.005 | <0.001 | 0.00926 | Yes | <0.001 | <0.001 |
| Ch4 | -0.010 | -0.015 | -0.005 | <0.001 | 0.01111 | Yes | <0.001 | <0.001 |
| Hippocampus HATA | -0.011 | -0.017 | -0.004 | 0.001 | 0.01296 | Yes | <0.001 | <0.001 |
| Ch123 | -0.008 | -0.013 | -0.002 | 0.009 | 0.01481 | Yes | <0.001 | <0.001 |
| Amygdala LB | -0.023 | -0.040 | -0.005 | 0.012 | 0.01667 | Yes | <0.001 | 0.001 |
| Hippocampus CA3 | -0.006 | -0.012 | -0.001 | 0.031 | 0.01852 | No | <0.001 | <0.001 |
| Hippocampus CA2 | -0.006 | -0.013 | 0.000 | 0.058 | 0.02037 | No | 0.004 | <0.001 |
| PSC 1 | -0.005 | -0.011 | 0.001 | 0.108 | 0.02222 | No | <0.001 | <0.001 |
| Broca BA44 | -0.004 | -0.010 | 0.001 | 0.120 | 0.02407 | No | 0.001 | <0.001 |
| Auditory Te1 2 | -0.005 | -0.011 | 0.001 | 0.133 | 0.02593 | No | <0.001 | <0.001 |
| Motor 4A | -0.004 | -0.010 | 0.001 | 0.133 | 0.02778 | No | <0.001 | <0.001 |
| Broca BA45 | -0.004 | -0.010 | 0.002 | 0.154 | 0.02963 | No | <0.001 | <0.001 |
| Hippocampus DG | -0.003 | -0.008 | 0.002 | 0.194 | 0.03148 | No | <0.001 | <0.001 |
| Hippocampus CA1 | -0.003 | -0.008 | 0.003 | 0.310 | 0.03333 | No | <0.001 | <0.001 |
| PSC 3a | 0.003 | -0.004 | 0.009 | 0.403 | 0.03519 | No | 0.102 | <0.001 |
| PSC 2 | -0.002 | -0.008 | 0.004 | 0.465 | 0.03704 | No | 0.003 | <0.001 |
| Hippocampus Subc | -0.002 | -0.006 | 0.003 | 0.525 | 0.03889 | No | <0.001 | <0.001 |
| Motor 4P | 0.002 | -0.004 | 0.008 | 0.579 | 0.04074 | No | 0.013 | <0.001 |
| Auditory Te1 0 | -0.005 | -0.025 | 0.016 | 0.654 | 0.04259 | No | 0.012 | 0.001 |
| hOC2 | -0.001 | -0.007 | 0.005 | 0.726 | 0.04444 | No | <0.001 | <0.001 |
| PSC 3b | -0.001 | -0.007 | 0.005 | 0.729 | 0.04630 | No | 0.005 | <0.001 |
| hOC1 | -0.001 | -0.007 | 0.005 | 0.785 | 0.04815 | No | <0.001 | <0.001 |
| Auditory Te1 1 | -0.001 | -0.010 | 0.008 | 0.807 | 0.05000 | No | <0.001 | <0.001 |

**Supplementary Table 5**. Slope parameter estimates of the regression of years since PD diagnosis onto standardized grey matter density for all PD.
